# The effectiveness of surgical management in knee flexion contracture in primary total knee arthroplasty

**DOI:** 10.12669/pjms.42.4.13311

**Published:** 2026-04

**Authors:** Muhammad Aamir, Noor Rahman, Faizan Shah, Waseem Khan

**Affiliations:** 1 Muhammad Aamir, Department of Orthopedics & Spine Surgery, Hayatabad Medical Complex, Peshawar, Pakistan; 2 Noor Rahman, Department of Orthopedics & Spine Surgery, Hayatabad Medical Complex, Peshawar, Pakistan; 3 Faizan Shah, Department of Orthopedics & Spine Surgery, Hayatabad Medical Complex, Peshawar, Pakistan; 4 Waseem Khan, Department of Orthopedics & Spine Surgery, Hayatabad Medical Complex, Peshawar, Pakistan

**Keywords:** Arthroplasty, Contracture, Knee Joint, Knee arthroplasty, Osteoarthritis, Total Knee Replacement

## Abstract

**Objectives:**

This study aimed to compare knee flexion contracture before & after Total knee arthroplasty (TKA) post-intraoperative soft tissue release, posterior osteophytes removal & distal femoral additional cutting.

**Methodology:**

This descriptive study was conducted in the department of Orthopedic & Spine Hayatabad Medical Complex, Peshawar from 1^st^ February 2023 to 30^th^ April 2025. The acquired sample size was 72. The inclusion criteria included all adult patients having age > 45years & having moderate to severe (Grade-II and III) flexion contracture who were undergoing primary total knee arthroplasty (TKA) and had either osteoarthritis or rheumatoid arthritis. Patients having mild flexion deformities such as Grade-I flexion contractures, and those with hip flexion contractures due to other conditions including psoriatic arthritis, haemophilic arthritis, or post-traumatic arthritis were excluded.

**Results:**

Out of 72 patients included in our study, 41(56.9%) were women & 33(45.8%) were men. 49(68%) patients had age <65years & 23(31.9%) patients had age >65years. The mean flexion contracture in pre-op patients was 16.9 degrees & in post-op patients mean flexion contracture was 5.9 degrees at one month of follow up, 5.5 degree at three months & 5.3 degree at six months of follow up.

**Conclusion:**

Knee flexion contracture is one of the debilitated conditions associated with knee OA & can be corrected in Total Knee Arthroplasty with soft tissue releases, posterior osteophytes removal & capsular release in moderate flexion contracture cases while flexion contracture >30º require additional resection of distal 2-3mm of distal femur.

## INTRODUCTION

Osteoarthritis is one of the prevalent & debilitating medical condition, that has a significant impact on an individual quality of life & can cause fatigue, pain, impaired sleep or disability.^1^ With an estimated 365 million cases worldwide, knee osteoarthritis (OA) is the most prevalent joint afflicted, affecting a sizable fraction of the population, according to the World Health Organization (WHO).^2^

Knee flexion contracture is one of the common deformities associated with advanced osteoarthritis of knee. Knee contractures impair mobility, raise energy costs, raise the chance of falls, and put additional strain on the hip and lumbar spine. Unilateral knee flexion contracture can also cause Limb length discrepancy.^3^

When a patient has a knee flexion contracture, their quadriceps must contract in order to keep their knee from slipping out of flexion when they should be bearing weight. In one study it was found that increasing flexion contracture from 15 to 30 resulted in a 22–51% increase in quadriceps workload. When walking with flexion contracture, the foot remains flat on the ground and the step is not rolled from heel to toe.^4^

Long-term flexion contracture causes tightening of the posterior capsule, parts of the collateral ligament, the gastrocnemius & the hamstrings, and the bony changes includes the loss of posterior aspect of the tibial plateau and erosion of the posterior femoral condyle.^5^

Flexion contracture is an issue with the extension space, and raising the extension space’s inadequate height to match the flexion space’s is challenging.^6^ Kinhoshita et al.^7^ found that the primary cause of postoperative flexion contracture was preoperative flexion contracture.

Flexion contractures need to be addressed both operatively & post-operatively with physiotherapy & rehabilitation. Numerous empirical surgical algorithms, such as ligament release, posterior osteophyte removal, posterior capsule release and further distal femoral resection, have been proposed.^8^

It is estimated that between 1.4 and 17% of patients develop flexion contracture following total knee arthroplasty. Many authors agree that flexion contracture particularly at 15 degrees or more, dramatically impairs functional performance and walking ability.^9^

Kim et al.^10^ in their study from South Korea found that the average flexion contracture angle difference between the starting and post-medial release angles was 5.2° ± 2.8°. Flexion contracture mean difference angle between the posterior cruciate ligament (PCL) release and the medial release step was 2.5° ± 2.2°. After routine bone cutting, the mean difference in flexion contracture angle was 3.1° ± 3.2° from the PCL release step.^11^

Umre et al.^9^ in their study from Nagpur, India operated 43 patients having mild to moderate fixed flexion deformity & stepwise followed algorithm which included soft tissue release, removal of posterior osteophytes, posterior capsule release, horizontal capsule release, gastrocnemius release & hamstring tenotomy.

Unawareness about the knee ROM & Knee flexion contracture after soft tissue balancing, posterior osteophytes removal & posterior capsular release was our research problem. Our research will guide surgeons about algorithm for removal of varying degree of flexion contracture in knee.

The objective of this study was to compare knee flexion contracture before & after Total knee arthroplasty (TKA) post-intraoperative soft tissue release, posterior osteophytes removal & distal femoral additional cutting.

## METHODOLOGY

This descriptive study was conducted in the department of Orthopedic & Spine from 1^st^ February 2023 to 30^th^ April 2025. Patients or attendants gave their informed consent for study. Using the Rao soft sample size calculator, the sample size was determined. Having margin of error 5%, the expected proportion was 4.9%, and the confidence interval was 95%. The required sample size was 72.

### Ethical Approval:

The study was approved by the hospital’s ethical committee on July 15, 2025 & approval number was 2770.

A non-probability consecutive sampling strategy was utilized for sampling. Lombardi classification system was utilized to classify flexion contractures; Grade-I (mild, less than 10 degrees), Grade-II (moderate, 10-30 degrees), and Grade-III (severe, greater than 30 degrees).

### The inclusion & Exclusion criteria:

It included all adult patients having age > 45years & having moderate to severe (Grade-II-III) flexion contracture who were undergoing primary total knee arthroplasty (TKA) and had either osteoarthritis or rheumatoid arthritis. Patients having mild flexion deformities such as Grade-I flexion contractures, and those with hip flexion contractures due to other conditions including psoriatic arthritis, hemophilic arthritis, or post-traumatic arthritis were excluded.

In order to measure the degree of FC, the patient was asked to completely extend their knees while lying supine and resting their ankle on a foam roller before to surgery & at one month, three months & six months post-surgery. The degree of FC was measured using a goniometer, which had its upper arm pointed towards the greater trochanter and its fulcrum over the lateral femoral epicondyle while lower arm pointing towards the lateral malleolus.

Moderate degree flexion contractures i.e., 10-30º were managed with soft tissue releases, posterior osteophytes removal & capsular release. Flexion contracture >30º required additional resection of distal 2-3mm of distal femur. Few patients having flexion contracture > 45-50º were managed with two staged procedures. In 1^st^ stage Manipulation under anesthesia was done followed by casting for two weeks. In 2^nd^ stage TKA surgery was performed & and the algorithm described above was used to execute the procedure according to the extent of knee contracture.

All patients fulfilling inclusion criteria were included in our study. Our demographic variables were the patient’s age (<65 years or more than 65 years) and gender (male or female) while presence of flexion contracture was our research variable. Patients were divided into two groups; all pre-operative patients having flexion contracture >10 degrees & fulfilling inclusion criteria were included in Group-A while post-operative patients, managed according to aforementioned algorithm while performing TKA were included in Group-B.The degree of flexion contracture was measured using goniometer & all Collection procedures were carried out under the direction of an Orthopaedics professor and associate professor with at least 15 years of post-fellowship experience and a qualified staff nurse.

### Statistical Analysis:

Data was analyzed using IBM-SPSS-V.25. The mean difference between flexion contracture of two groups was measured using paired t-test which compare the mean flexion contracture before & after TKA.

## RESULTS

Out of 72 patients included in our study, 41(56.9%) were women & 33(45.8%) were men. 49(68%) patients had age <65years & 23(31.9%) patients had age >65years. All patients underwent Total Knee Replacement for knee osteoarthritis & for flexion contracture release aforementioned algorithm was applied.

The mean flexion contracture in pre-op patients included in Group-A was 16.9 degrees. Sixty seven patients were included in Lombardi Grade-II (moderate, 10-30 degrees) & 5 patients in Grade-III (severe, greater than 30 degrees). The mean flexion contracture in post-op patients included in Group-B was 5.9 degrees at one month of follow up, 5.5 degree at three months & 5.3 degree at six months of follow up.

**Table-I:** Paired t-test to compare flexion contracture of two groups at one months of follow up.

	*N*	*Mean*	*Standard Deviation*	*95% confidence interval of difference*		*t-value*	*Degree of freedom*	*p-value*
				*Lower*	*Upper*	13.300	71	0.0001
Group-A	72	16.979	5.5433	9.4159	12.7369			
Group-B	72	5.90	5.329					

The mean difference between flexion contracture of two groups was measured using paired t-test at one month, three months & six months. The results were statistically significant i.e., p-value<0.05 showing effectiveness of aforementioned surgical procedures to be effective in treating flexion contracture in TKR patients.

DISCUSSION

In our study it was found that post Total knee replacement using soft tissue releases, posterior osteophytes removal & capsular release for 10–30 degrees flexion contracture & additional resection of distal 2-3mm of distal femur for contracture >30 degrees, mean flexion contracture was improved from 19.79 to 5.90 at one month, 5.56 at two months & 5.35 at three months.

Our results showed improvement in flexion contracture post TKA & the results were statistically significant as well i.e., p-value <0.05. Similar to our study Umre et al.^9^ in their study from Nagpur, India operated 43 patients having mild to moderate fixed flexion deformity & stepwise followed algorithm which included soft tissue release, removal of posterior osteophytes, posterior capsule release, horizontal capsule release, gastrocnemius release & hamstring tenotomy. At one-year follow-up, the pre-operative mean range of motion (ROM) improved from 71.37 ± 22.18 to 107.7 ± 10.38.

Chai et al.^12^ in their study conducted on 32 patients having pre-operative mean flexion contracture 37.69 ± 11.79°, was improved to post-operative 5.78 ± 4.44° (p < 0.001) using frontal ligament balance following the removal of osteophytes, further distal femur resection up to 4-mm, and posterior capsule fusiform capsulectomy.

Similar to our study Bellemans et al.^13^ in their study found that for a flexion contracture <30 degrees an algorithm consisting of balancing the mediolateral ligament by removing all osteophytes and over resecting the distal femur by 2 mm & gradual relaxation of the posterior capsule and gastrocnemius, correction can be achieved while for a flexion contracture >30 degrees, 17 patients had achieved correction after these mentioned steps, while 10 patients required additional resection of distal femur to achieve correction & eight patients required tenotomy of flexor tendons including hamstring tendon release to achieve correction. At end of surgery two patients had a flexion contracture between 10-15 degrees & two patients had developed peroneal nerve palsy one of which was permanent.

**Table-II:** Paired t-test to compare flexion contracture of two groups at three months of follow up.

	*N*	*Mean*	*Standard Deviation*	*95% confidence interval of difference*		*t-value*	*Degree of freedom*	*p-value*
				*Lower*	*Upper*	13.551	71	0.0001
Group-A	72	16.979	5.5433	9.7427	13.1045			
Group-B	72	5.56	5.474					

**Table-III:** Paired t-test to compare flexion contracture of two groups at six months of follow up

	*N*	*Mean*	*Standard Deviation*	*95% confidence interval of difference*		*t-value*	*Degree of freedom*	*p-value*
				Lower	Upper	13.781	71	0.0001
Group-A	72	16.979	5.5433	9.9489	13.3150			
Group-B	72	5.35	5.460					

In one study it was found that the extension space can be efficiently widened and flexion contracture corrected by loosening the posterior capsule surrounding the intercondylar notch.^14^ Smith et al.^15^ in their study found that for every 2-mm of bone resected, approximately 3.6 of the extension was restored while Liu et al.^16^ determined that 3.36º was returned with the first 2 mm of distal femur resection, whereas Matziolis et al.^17^ discovered a linear relationship of 2.2º ±0.3º of regained extension done per millimeter of femoral resection.

Kim et al.^10^ in their study from South Korea found that the average flexion contracture angle difference between the starting and post-medial release angles was 5.2° ± 2.8°. Flexion contracture mean difference angle between the posterior cruciate ligament (PCL) release and the medial release step was 2.5° ± 2.2°. After routine bone cutting, the mean difference in flexion contracture angle was 3.1° ± 3.2° from the PCL release step.

Chalmers et al.^18^ in their study found that mean coronal laxity increased by a mean of 3.1° (SD 0.18°) (p < 0.001) and 2.7° (SD 0.30°) (p < 0.001) with + 2 mm resection at 30° and 45° of flexion, respectively. At 30° and 45° of flexion, mean coronal laxity increased by 6.5° (SD 0.56°) (p < 0.001) and 5.5° (SD 0.72°) (p < 0.001), respectively, with +4 mm resection.

Leie et al.^19^ in their study found that osteophyte size and the degree of extension corrected at the time of surgery showed a positive, linear connection (Pearson correlation 0.327, p value<0.05). Extension increases by 2.7° when Grade-2 or Grade-3 osteophytes are removed, respectively.

So, in literature a variety of different algorithms are devised for the treatment of flexion contractures, which includes soft tissue releases & bony resection for severe deformities correction. Our study provides a comprehensive & step wise approach in the treatment of flexion contracture, aimed at releasing moderate to severe flexion contractures.

### Limitations:

In our study we did not perform CT-scan or MRI in radiologic assessment of flexion contracture, as they may show bone loss or posterior osteophytes in more detail.

## CONCLUSION

Knee flexion contracture is one of the debilitated conditions associated with knee OA & can be corrected in Total Knee Arthroplasty with soft tissue releases, posterior osteophytes removal & capsular release in moderate flexion contracture cases while flexion contracture >30º require additional resection of distal 2-3mm of distal femur.

### Authors Contribution:

**MA:** Designed, did statistical analysis & editing of manuscript, is responsible for integrity of research. **NR, FS, WK:** Did data collection, statistical analysis and manuscript writing. All authors have approved the final manuscript for publication.
